# Association Between Exercise and Brain‐Derived Neurotrophic Factor (BDNF) Levels in Diabetes Mellitus: A Systematic Review and Meta‐Analysis

**DOI:** 10.1155/jdr/5163895

**Published:** 2026-10-21

**Authors:** Rumeesa Parveen, M. Abdur Rahman Aizaz, Sarah Sohail, Sara Jawaid, Abdullah Amjad, Syeda Sana-e-Zehra Naqvi, Dirha Amjad, Araj Naveed Siddiqui, Asma Azam, Laiba Shamim, Umaimah Naeem, Sakan Binte Imran

**Affiliations:** ^1^ Jinnah Sindh Medical University Karachi Pakistan jsmu.edu.pk; ^2^ Services Institute of Medical Sciences Lahore Pakistan sims.edu.pk; ^3^ Quaid-e-Azam Medical College Bahawalpur Pakistan qamc.edu.pk; ^4^ Karachi Metropolitan University Karachi Pakistan; ^5^ Sir Salimullah Medical College Dhaka Bangladesh

## Abstract

**Background:**

Diabetes mellitus (DM) is a metabolic disorder characterized by a consistently elevated blood sugar level. DM poses a major risk factor for many neurodegenerative diseases, causing a decline in cognitive function. BDNF (brain‐derived neurotrophic factor) is a neurotrophin in the central and peripheral nervous systems that plays a crucial role in the development of brain circuits. Hence, disturbance in levels of BDNF is linked to the occurrence of neurodegenerative diseases and cognitive decline in diabetic patients. Physical exercise has emerged as a nonpharmacological intervention in elevating BDNF levels. Our systematic review and meta‐analysis evaluate the relationship between a globally affecting metabolic disorder, diabetes, with decreased BDNF levels, and explore exercise as a promising potential intervention.

**Methods:**

After screening 37 articles, 4 studies have been included in the systematic review and meta‐analysis, out of which 3 of them are randomized controlled trials (RCTs), and 1 is a crossover trial. The search was conducted on PubMed (MEDLINE), ScienceDirect, Embase, Scopus, and Cochrane Central databases from inception to June 2025. PRISMA (Preferred Reporting Items for Systematic Reviews and Meta‐Analyses) guidelines were followed to conduct a systematic review. Cochrane risk‐of‐bias tool was used to assess the quality of the studies. Data were pooled using a random effect model. The protocol for this systematic review was registered in PROSPERO.

**Results:**

A total of 37 articles were screened, 3 RCTs and 1 crossover trial with 131 participants were included in the meta‐analysis. Serum BDNF levels were higher in the exercise group compared with controls (MD = 73.50; *p* = 0.05). A small reduction in body fat percentage in the exercise group compared with the control was found (MD = −1.24*%*; *p* = 0.17), which was not statistically significant. Body weight and fasting blood glucose (FBG) showed no statistically significant differences between the exercise and control groups (MD = 0.97; *p* = 0.65), (MD = −27.20; *p* = 0.31). Moderate to high heterogeneity was noticed across the data analyzed.

**Conclusion:**

This meta‐analysis overall concludes that regular exercise positively affects serum BDNF levels in diabetics. However, the small number of trials and small sample sizes has limited the conclusions. Further large‐scale RCTs are required to determine the long‐term neurological effects of exercise in diabetics.

## 1. Introduction

Diabetes mellitus is a metabolic disorder caused by deficient insulin levels or resistance and is characterized by hyperglycemia and hyperlipemia [1]. It is also correlated with several other cardiovascular and microvascular comorbidities, such as hypertension, ischemic heart disease, obesity, dyslipidaemia, diabetic retinopathy, nephropathy, and neuropathy [2, 3]. Diabetes poses a global challenge as it decreases the average life expectancy by 6 years [4], and statistics show that 537 million people were affected by diabetes in 2021, and this figure would soar to 783 million by 2045 [5].

Neurotrophins play an important role in neuronal development, function, and survival [6]. Of the four neurotrophins, brain‐derived neurotrophic factor (BDNF) is synthesized as a glycosylated precursor molecule named pre‐proBDNF in the central and peripheral nervous system. It works via TrkB receptor activation [7]. BDNF plays a crucial role in developing brain circuits and architecture, formation and maintenance of neurons, and synaptic and neuronal network plasticity. Since it is also involved in forming memory and learning processes, it becomes imperative to understand that the physical manifestation due to disturbance in BDNF levels is linked to the occurrence of neurodegenerative and psychiatric diseases, like Alzheimer′s disease, and major depressive disorder [8]. Moreover, people suffering from major depressive disorder are also likely to exhibit lower peripheral levels of BDNF [9]. This makes one easily understand what relevance BDNF has to diabetes and what its ultimate result could be.

Continuing in this regard, BDNF levels are found to improve due to exercise, and greater intensities of exercise increase BDNF levels significantly [10], and that BDNF plays a vital role in cognitive tasks is well evaluated [11]. Similarly, it is also found that aerobic exercise can be an impactful intervention for the improvement of BDNF‐dependent executive functioning and works better than cognitive stimulation alone [12]. This meta‐analysis discusses that physical exercise, if applied as an intervention, would lead to an increase in BDNF levels in neurodegenerative disorders [13] and will ultimately improve neuroplasticity and cognitive benefit. Our meta‐analysis hence evaluates the relationship between a globally affecting, burdening metabolic disorder, diabetes, with decreased BDNF levels, and explores exercise as a promising potential intervention that could correct the BDNF levels.

We found that the existing literature is rich in meta‐analyses that are based on evaluating BDNF levels in only one particular type of diabetes, and a research gap exists as no particular meta‐analysis examines different types of diabetes jointly. However, to our knowledge, our meta‐analysis is the only one that has included different types of diabetes to date, evaluating and providing comprehensive results altogether, providing a concrete base for further research as it proposes further useful suggestions for better research and discovering more promising outcomes. Alongside this, when recent pool of meta‐analyses is evaluated, it could be inferred that our meta‐analysis and systematic review not only updates the literature on this topic with recent RCTs, but also has an added benefit of including a broader range of age groups adolescents (15.6 years) to older adults (63.2 years), and is the first to include neuromuscular electrical stimulation (NMES) as an exercise intervention when compared with the latest meta‐analysis and systematic review [14]. Back in 2018, as demonstrated by this trial, NMES improved BDNF levels [15]. This provides a strong basis for further research into NMES as an alternative intervention for patients who may be unable to perform exercise voluntarily.

## 2. Methods

### 2.1. Study Design

This systematic review and meta‐analysis was conducted according to the Preferred Reporting Items for Systematic Reviews and Meta‐Analyses (PRISMA 2020) guidelines. The study protocol was registered in the International Prospective Register of Systematic Reviews (PROSPERO) (Registration No. CRD420251116372) before study selection.

### 2.2. Data Sources and Search Strategy

A comprehensive literature search was conducted in PubMed (MEDLINE), Scopus, ScienceDirect, Embase, and the Cochrane Central Register of Controlled Trials (CENTRAL) from the earliest available records through June 2025. Additionally, ClinicalTrials.gov was searched for ongoing or unpublished studies, and the reference lists of all eligible articles and relevant systematic reviews were manually screened to identify additional studies.

The search strategy was formulated according to the PICO framework and combined both controlled vocabulary (MeSH in PubMed and Emtree in Embase) with keywords. The main search terms included Diabetes Mellitus, Exercise, Control Groups, and Brain‐Derived Neurotrophic Factor (BDNF).

The core PubMed search strategy was as follows:

(“Diabetes Mellitus”[Mesh] OR diabetes mellitus) AND (“Exercise”[Mesh] OR exercise OR physical exercise OR aerobic exercise OR resistance training OR combined exercise OR neuromuscular electrical stimulation OR NMES) AND (“Control Groups”[Mesh] OR control OR sedentary control OR placebo OR no intervention OR detraining) AND (“Brain‐Derived Neurotrophic Factor”[Mesh] OR brain‐derived neurotrophic factor OR BDNF).

Equivalent Emtree terms and database‐specific syntax were applied for Embase, whereas appropriate subject headings and keywords were adapted for Scopus, ScienceDirect, and CENTRAL (File S1).

### 2.3. Eligibility Criteria (PICO)

The eligibility criteria were predefined according to the PICO framework.

• Population (P): Men and women diagnosed with diabetes mellitus, including Type 1 diabetes mellitus, Type 2 diabetes mellitus, and gestational diabetes mellitus.
• Intervention (I): Exercise training interventions, including aerobic exercise, resistance training, combined aerobic and resistance training, and NMES.
• Comparison (C): Control groups receiving no exercise intervention, sedentary lifestyle/usual care, placebo or sham intervention, and detraining (exercise withdrawal) where applicable.
• Outcomes (O): The primary outcomes are BDNF and fasting blood glucose (FBG). The secondary outcomes are body weight, body mass index (BMI), percent body fat (PBF), waist circumference, glycated hemoglobin (HbA1c), insulin resistance, and cardiovascular parameters (blood pressure and VO_2_peak).

Only randomized controlled trials (including randomized crossover trials) involving human participants were included. Case reports, observational studies, cross‐sectional studies, conference abstracts, reviews, editorials, letters, expert opinions, and animal studies were excluded.

### 2.4. Study Selection

Extracted citations were imported into EndNote for duplicate removal. Two reviewers screened titles and abstracts independently, followed by full‐text assessment of potentially eligible studies. Disagreements were resolved through discussion, and when agreement could not be reached, a third reviewer independently evaluated the study and made the final decision. For studies with missing or incomplete data, the corresponding authors were contacted where feasible to obtain the required information. Studies with unresolved missing essential outcome data were excluded from full‐text eligibility assessment.

### 2.5. Data Extraction and Risk of Bias Assessment

Two reviewers separately extracted study characteristics using a standardized Microsoft Excel spreadsheet. Extracted variables included study characteristics, participant demographics, intervention details, comparator characteristics, duration of follow‐up, and outcome measures. Because BDNF concentrations were reported in different units (e.g., pg/mL and ng/mL), all values were converted to a common unit before quantitative synthesis (1 ng/mL = 1000 pg/mL).

Methodological quality was independently assessed by two reviewers using the Cochrane Risk of Bias Tool Version 2 (RoB 2). Studies were classified as having low risk of bias, some concerns, or high risk of bias. Any disagreements were resolved by discussion with a third reviewer until agreement was achieved. The certainty of the evidence for each outcome was assessed using the Grading of Recommendations Assessment, Development and Evaluation (GRADE) approach.

### 2.6. Statistical Analysis

Statistical analyses were performed using Review Manager (RevMan) Version 5.4. Continuous outcomes were pooled using mean difference (MD) with 95% confidence intervals (CIs) because all studies reported BDNF and FBG using the same measurement scales after unit standardization. Therefore, the use of MD was considered more appropriate than standardized MD, allowing direct interpretation of treatment effects. Random‐effects models were used to account for anticipated clinical and methodological heterogeneity. Statistical heterogeneity was evaluated using the *I* ^2^ statistic, with values < 50%, 50%–75%, and > 75% representing low, moderate, and substantial heterogeneity, respectively. One included study used a randomized crossover design. To avoid unit of analysis errors, only data from the first treatment period before crossover were included in the meta‐analysis, in accordance with Cochrane recommendations. Leave‐one‐out sensitivity analyses were performed by sequentially excluding each individual study to assess the influence of individual studies on the pooled effect estimate and between‐study heterogeneity. Forest plots were generated to visually summarize the pooled effects.

## 3. Results

### 3.1. Study Selection

A total of 1288 articles were identified in the literature search, which included RCTs and observational studies. After removing duplicates, 828 articles were left for title and abstract screening. Based on reviewing by title and abstract, 38 reports were sought for full‐text screening, out of which 8 reports were excluded due to the unavailability of the full text. Twenty six studies were excluded because they did not meet the eligibility criteria (reasons: wrong study design [*n* = 4], no BDNF outcome reported [*n* = 6], no exercise intervention [*n* = 5], no control group [*n* = 7], and wrong population [*n* = 4]). Four studies were included in the current systematic review and meta‐analysis, out of which 3 are RCTs, and 1 is a randomized crossover‐controlled trial for which the date of evaluation is taken before the carryover period. The flow diagram of the study selection process is presented in Figure 1.

**Figure 1:**
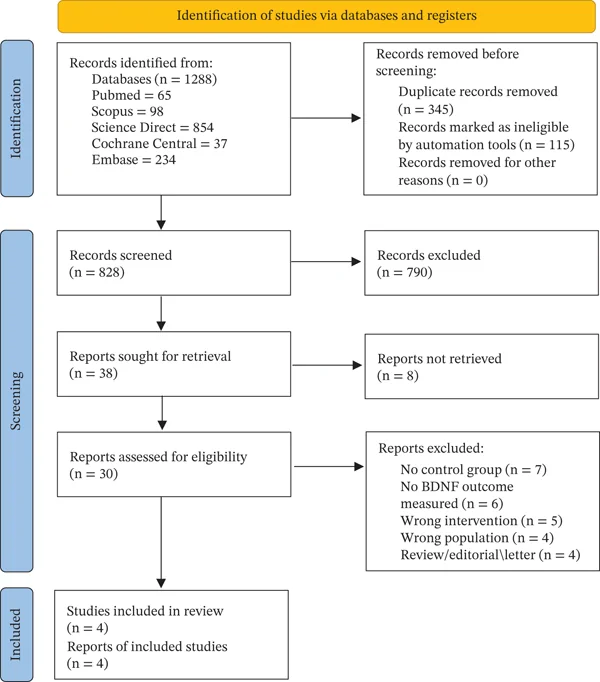
Preferred Reporting Items for Systemic Reviews and Meta‐Analysis (PRISMA) flow diagram.

### 3.2. Study Characteristics

We identified four studies, which presented diverse baseline characteristics, including age, race, sex, weight, height, and serum BDNF level. A total of 131 participants were analyzed in the present review with a mean age of 60.8 years. Three studies included only men, and 1 study included both men and women. Seventy‐four out of 131 received interventions of NMES, aerobic exercise, resistance training, or combined aerobic and resistance training. The time period for the exercise training ranged between 8 weeks and 9 months, and training frequency varied between 3 and 5 sessions per week. The general characteristics of the studies included in this meta‐analysis are given in Table 1.

**Table 1:** Baseline characteristics of the RCTs included in the systematic review and meta‐analysis. Data are presented as mean ± SD.

Study (author, year)	Toshiaki et al. 2018 [16]	Swift et al. 2012 [17]	Donyaei et al. 2024 [18]	Sung Soo Lee et al. 2014 [19]
Study design	RCT (crossover trial)	RCT	RCT	RCT
Population	Type 2 DM	Type 2 DM	Type 2 DM	Juvenile obesity and Type 2 DM
Intervention	NMES	Aerobic, resistance, and combined training	Combined exercise training	Aerobic exercise
Control	Nonexercise control group	Nonexercise control group	Detraining (no exercise)	Control group
Time period of intervention (*n*)	8 weeks	9 months	12 weeks	12 weeks
No. of patients (*n*)	14	63	34	30
Intervention	6	40	17	15
Control	8	23	17	15
Age (years)				
Intervention	63.2 ± 3.0	54.2 ± 7.3	61.3 ± 5.7	15.57 ± 2.14
Control	63.2 ± 3.0	58.4 ± 8.7	62.1 ± 5.1	16.45 ± 1.36
Sex (%)				
Female	100	61.9	100	34.6
Male	0	38.1	0	65.4
Race % (*n*)				
White (intervention/control)	NR	62.5 (25)/52.2 (12)	NR	NR
Black (intervention/control)	NR	37.5 (15)/43.5 (10)	NR	NR
Other (intervention/control)	NR	0 (0)/4.4 (1)	NR	NR
BMI (kg/m^2^)				
Intervention	27.0 ± 1.2	33.9 ± 6.4	30.2 ± 1.3	23.72 ± 4.47
Control	27.0 ± 1.2	33.3 ± 6.3	29.9 ± 1.2	22.35 ± 3.94
Height (cm)				
Intervention	NR	NR	158.7 ± 4.2	161.15 ± 7.70
Control	NR	NR	157.1 ± 5.2	171.11 ± 5.17
Weight (kg)				
Intervention	76.1 ± 3.5	97.1 ± 19.9	75.8 ± 4.9	61.84 ± 13.68
Control	76.1 ± 3.5	93 ± 19.4	73.7 ± 6.1	65.92 ± 14.58
Body fat (%)				
Intervention	NR	36 ± 7.7	NR	28.20 ± 4.77
Control	NR	37.2 ± 6.9	NR	16.56 ± 6.96
Serum BDNF (pg/mL)				
Intervention	NR	NR	18,870.4 ± 8153.5	25,136.14 ± 3814.16
Control	NR	NR	16,928.1 ± 6781.7	21,178.33 ± 4306.14
HbA1c (%)				
Intervention	7.7 ± 0.2	7.2 ± 1.1	8.09 ± 0.73	NR
Control	7.7 ± 0.2	7.8 ± 1.7	8.22 ± 0.79	NR
Fasting Glucose (mg/dL)				
Intervention	NR	NR	161.41 ± 15.11	NR
Control	NR	NR	170.17 ± 14.8	NR
Waist circumference (cm)				
Intervention	NR	109.6 ± 14.7	0.966 ± 0.02	NR
Control	NR	106.9 ± 13.3	0.963 ± 0.01	NR
Diabetes duration (years)				
Intervention	9.6 ± 1.4	7.9 ± 6.0	NR	NR
Control	9.6 ± 1.4	7.0 ± 5.4	NR	NR
Systolic blood pressure (mmHg)				
Intervention	NR	123 ± 11	171.5 ± 10.8	NR
Control	NR	124.7 ± 13.9	172.1 ± 8.02	NR
Diastolic blood pressure (mmHg)				
Intervention	NR	76.2 ± 9.4	105.8 ± 1.9	NR
Control	NR	75.5 ± 7.8	106.05 ± 2.4	NR
Absolute VO2peak (L/min)				
Intervention	NR	2.0 ± 0.5	NR	33.28 ± 3.76
Control	NR	1.7 ± 0.5	NR	28.60 ± 4.88

Abbreviations: HbA1c, glycated hemoglobin; NR, not reported; RCT, randomized controlled trial; SD, standard deviation; T2DM, Type 2 diabetes mellitus.

Regarding the sex distribution shown in Table 1, the study by Toshiaki et al. [16] consisted of 100% male participants, and the study by Donyaei et al. [18] consisted of 100% female participants. Swift et al. [20] reported 61.9% female and 38.1% male participants; Sung Soo Lee et al. [19] reported 34.6% female and 65.4% male participants. This heterogeneity in sex distribution means that pooled results should be interpreted with caution.

### 3.3. Study Outcomes

Primary outcomes were changes in serum BDNF levels and FBG. RCTs that provided clear and measurable serum BDNF data in units (pg/mL or ng/mL) were included. Data extraction was performed at baseline, postintervention, and follow‐up time points. Secondary outcomes were FBG, PBF, homeostatic model assessment (HOMA) (mainly HOMA‐IR) as a marker of insulin resistance, body fat mass and body weight. FBG was reported in either mg/dL or mmol/L and was converted to mmol/L for pooling (conversion factor: 1 mmol/L = 18.018 mg/dL). PBF was expressed as a percentage of total body weight. Body fat mass and body weight were recorded in standard units. For each outcome, data were extracted at preintervention and postintervention (and follow‐up when reported) and pooled using the MDs with 95% CI. Standardized MD was not applied because all studies used the same assay type (enzyme‐linked immunosorbent assay [ELISA]) for the measurement of BDNF, and therefore absolute changes could be compared directly.

### 3.4. Risk of Bias Assessment

The quality of individual study analysis was assessed using the Cochrane Risk of Bias tool. The results of risk bias assessment are presented in Figure 2. The study by Donyaei et al. [18] was judged to have a low risk of bias across all domains, including the randomization process, deviations from intended interventions, missing outcome data, measurement of outcomes, and selection of reported results, resulting in an overall low risk of bias. Similarly, the study by Toshiaki et al. [16] was assessed as low risk in all domains and was therefore considered to have an overall low risk of bias. The study by Swift et al. [17] showed some concerns in the domain of deviations from intended interventions, whereas all other domains were rated as low risk, leading to an overall judgment of some concerns. In contrast, the study by Sung Soo Lee et al. [19] demonstrated a high risk of bias in the randomization process domain, although the remaining domains were assessed as low risk; consequently, this study was judged to have an overall high risk of bias.

**Figure 2:**
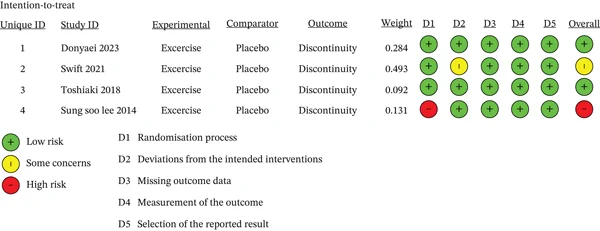
Risk of bias assessment of the RCTs evaluated using the Cochrane Risk of Bias Tool 2.

### 3.5. Study Evaluation

#### 3.5.1. BDNF Levels in the Exercise Versus Control Group

Serum BDNF levels were reported in four studies, which included a total of 131 participants (74 in the exercise group and 57 in the control group). Parallel‐group RCTs and crossover trials were analyzed as separate subgroups with a random‐effects model due to differences in study design. BDNF values reported in nanograms per milliliter were converted to picograms per milliliter (1 ng/mL = 1000 pg/mL) before pooling. Overall analysis of the pooled data revealed a modest increase in BDNF levels in the exercise group compared with controls (MD = 73.50 pg/mL, 95% CI: 0.43–146.58; *p* = 0.05), suggesting a borderline statistically significant effect that should be interpreted with caution. There was moderate heterogeneity (*I* ^2^ = 68*%*) across all studies, which reflects variability in estimates of effect. But heterogeneity within the parallel‐group RCT subgroup was low (*I* ^2^ = 39*%*) and no significant differences between crossover and parallel‐group study designs were seen in the subgroup analysis (*p* = 0.39). Overall, findings suggest a positive trend of increased BDNF levels with exercise, however, the borderline *p* value and moderate heterogeneity warrant cautious interpretation.

##### 3.5.1.1. Sensitivity Analysis

To test the robustness of the BDNF findings, we conducted leave‐one‐out sensitivity analyses to assess the influence of individual studies on the pooled estimate and heterogeneity (File S2). Exclusion of Donyaei 2023 reduced the heterogeneity from *I* ^2^ = 68*%* to *I* ^2^ = 42*%* and resulted in a change in the pooled effect estimate from MD 73.50 pg/mL (95% CI 0.43–146.58) to MD 995.30 pg/mL (95% CI −1174.93 to 3165.52). The statistical significance of the pooled effect was no longer maintained (*p* = 0.37), indicating that the overall finding was sensitive to the inclusion of this study (Figure 3).

**Figure 3:**
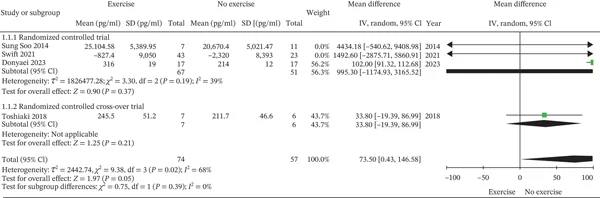
Forest plot illustrating the pooled effect of exercise interventions compared with control groups on serum BDNF levels in patients with diabetes mellitus. Values in bold highlight important results. Abbreviation: CI, confidence interval.

The pooled analysis of all four studies indicates a slightly positive increasing effect on BDNF levels in the exercise group compared with the control or nonexercise group (MD = 73.50; 95% CI: [0.43, 146.58]; *p* = 0.05; *z* = 1.97; *I* ^2^ = 68*%*).

#### 3.5.2. PBF in the Exercise Versus Control Group

The analysis of PBF included four studies. A total of 131 participants (74 in the exercise group and 57 in the control group) were included. Subgroup analysis was conducted because of the differences in study design. The pooled analysis overall indicated a small reduction in PBF in the exercise group compared with the control (MD = −1.24*%*, 95% CI: −3.01 to 0.53; *p* = 0.17), which was not statistically significant. The pooled analysis showed moderate heterogeneity (*I* ^2^ = 72*%*), which suggests variability in the effect estimates. There was high heterogeneity within the parallel‐group RCT subgroup (*I* ^2^ = 81*%*) and subgroup analysis found no significant difference between crossover and parallel group designs (*p* = 0.61) (Figure 4).

**Figure 4:**
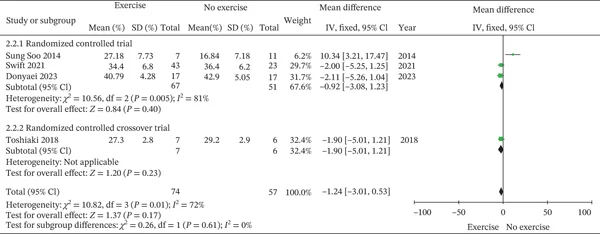
Forest plot comparing the effects of exercise interventions and control conditions on body fat percentage in individuals with diabetes mellitus. Values in bold indicate significant values representing the result. Abbreviation: CI, confidence interval.

The overall pooled analysis showed that the MD between the two groups was very small (−1.24%) and did not significantly affect the percentage of body fat in the exercising group (MD = −1.24; 95% CI: −3.01 to 0.53; *p* = 0.17; *z* = 1.37; *I* ^2^ = 72*%*).

#### 3.5.3. Body Weight in the Exercise Versus Control Group

Two studies reported this outcome, comprising a total of 47 participants, with 24 in the exercise group and 23 in the control group. The pooled analysis showed no statistically significant difference in body weight between the exercise and control groups (MD = 0.97 kg, 95% CI: −3.22 to 5.15; *p* = 0.65). Heterogeneity across the studies was low (*I* ^2^ = 0*%*), indicating consistent findings between studies (Figure 5).

**Figure 5:**
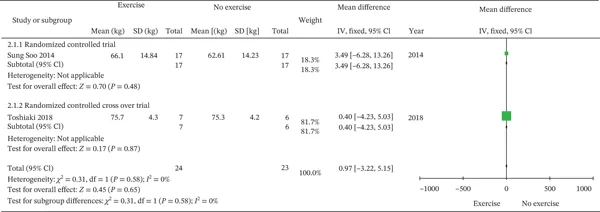
Forest plot showing the pooled effect of exercise interventions on body weight (kg) compared with control groups in patients with diabetes mellitus. Bold values are represent important results. Abbreviation: CI, confidence interval.

The MD (0.97 kg) shows a very small difference in favor of reduction of body weight between the two groups (MD = 0.97; 95% CI: [−3.22, 5.15]; *p* = 0.65; *z* = 0.45; *I* ^2^ = 0*%*). This suggests that there is no significant difference in weight loss between the two groups. The overall heterogeneity (*I* ^2^ = 0*%*) is low, indicating similar findings from both studies.

#### 3.5.4. FBG in the Exercise Versus Control Group

Two studies assessed FBG following exercise interventions. Both studies demonstrated a reduction in FBG in the exercise groups compared with the control groups; however, the magnitude of the effect differed considerably between studies (Donyaei et al.: MD = −5.36 mmol/L, 95% CI: −7.62 to −3.10; Toshiaki et al.: MD = −0.60 mmol/L, 95% CI: −1.02 to −0.18). This substantial discrepancy resulted in high heterogeneity (*I* ^2^ = 94*%*) (File S2). Given the small number of studies and the extreme heterogeneity, a pooled estimate was not considered sufficiently reliable, and the findings were therefore interpreted individually.

### 3.6. Publication Bias

Since the number of included studies was small (*n* < 10), publication bias could not be assessed by funnel plots or formal tests (e.g., Egger′s test). The possibility of publication bias cannot be excluded and should be considered when interpreting the results.

### 3.7. Summary of Findings and Certainty of Evidence

Figure 6 shows the main results and certainty of evidence. The certainty of evidence was assessed by using the GRADE approach. The evidence for BDNF, percentage of body fat and body weight was downgraded to moderate due to imprecision (borderline significance and wide CIs) and moderate heterogeneity. First, the certainty of evidence for FBG was rated high according to GRADE criteria. However, this high certainty rating should be interpreted cautiously due to extreme heterogeneity (*I* ^2^ = 99*%*), the small number of studies (*n* = 2), and the nonsignificant pooled effect. The inconsistency of FBG findings (large differences in effect magnitude across studies) considerably lowers confidence in the pooled estimate, and GRADE rating may overestimate evidence certainty. Further studies should validate FBG findings with more consistent results.

**Figure 6:**
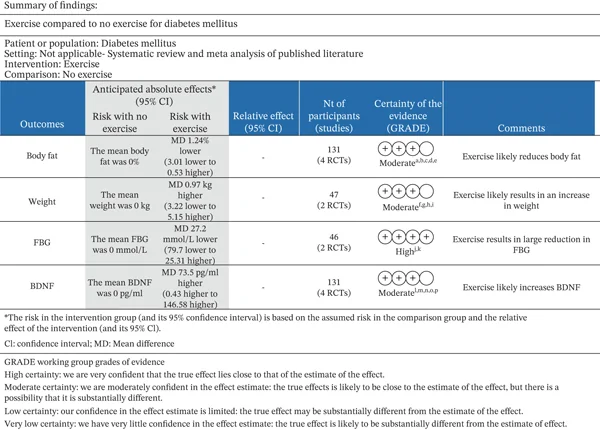
Certainty of evidence for each outcome and summary of findings table generated using GRADEpro, showing key outcomes (body fat, body weight, BDNF, FBG) and effect estimates. *Note:* The high certainty rating for FBG should be interpreted cautiously because of the very large heterogeneity (*I* ^2^ = 99*%*) and small number of studies included. Values in bold denote the significant results.

## 4. Discussion

Individuals with diabetes mellitus are susceptible to metabolic dysregulation, cardiovascular risk, and cognitive decline. It is a serious public health issue that needs to be effectively prevented and managed. Because it can mitigate the risks associated with a sedentary lifestyle, regular exercise is useful for adult individuals with diabetes who are sedentary [21–23].

Our pooled analysis suggests that exercise interventions may lead to modest increases in serum BDNF levels, supporting the hypothesis that physical activity can enhance neuroplasticity in individuals with diabetes. This is corroborated by observational and experimental data showing that elevated BDNF predicts better metabolic outcomes in long‐term illnesses including diabetes mellitus [24, 25].

This meta‐analysis is aimed at examining the impact of exercise training on BDNF levels in individuals with diabetes. A total of four studies were analyzed, comprising three RCTs and one crossover‐controlled trial. The RCTs employed various exercise modalities, including aerobic, resistance, combined aerobic–resistance training, and NMES. The meta‐analysis indicated that exercise interventions resulted in a modest yet beneficial increase in serum BDNF relative to controls (MD = 73.50 pg/mL; 95% CI: 0.43–146.58; *p* = 0.05). Three of the four trials demonstrated improvements in BDNF levels, whereas one reported a neutral effect. The overall pattern indicates consistent neurotrophic benefits associated with structured exercise, despite variability and inclusion of only a small number of studies. Similar findings of increased serum BDNF levels with exercise in patients with diabetes mellitus have been reported in previous meta‐analysis and systematic reviews [16–18, 26].

Despite individual studies demonstrating a significant decrease in FBG, findings by Donyaei et al. reported that improvements persisted beyond the intervention period, though diminished after detraining [19]. Although both included studies reported reductions in FBG, the magnitude of the effects differed markedly, resulting in extreme heterogeneity (*I* ^2^ = 99*%*). Therefore, these findings do not provide sufficient evidence to conclude that exercise significantly improves FBG. Two trials indicated reductions in insulin levels and enhanced insulin sensitivity, corroborating previous findings that exercise improves glucose uptake and insulin signaling via neurotrophic and skeletal muscle adaptations. Pooled findings indicated no significant effects on body weight or PBF (MD = −1.24*%*, 95% CI: −3.01 to 0.53; *p* = 0.17), aligning with evidence that neurotrophic and metabolic adaptations may occur independently of substantial weight loss.

NMES demonstrated reductions in FBG and body fat, along with increases in BDNF levels, though with little effect on HbA1c and lipids [27–29]. These results align with the findings of Donyaei et al. [18], which indicated that 12 weeks of combined aerobic and resistance training in women with T2DM significantly result in increased serum BDNF and facilitated improvements in depression, despite a decrease in BDNF levels after 8 weeks of detraining. Conversely, Swift et al. [17] found no significant changes in BDNF levels after 9 months of aerobic, resistance, or mixed training in the HART‐D trial (*n* = 150); however, reductions in waist circumference were positively correlated with BDNF variations [20]. These contradictory findings underscore the intricacy of BDNF regulation, affected by sex, adherence, initial body composition, and exercise intensity.

The overall findings indicate that both aerobic and resistance training, individually or in conjunction, can beneficially affect BDNF and metabolic health. Short‐term and closely monitored interventions (8–12 weeks) seem to have a bigger impact on BDNF, whereas long‐term programs may have problems with adherence and physiological adaptation, which could weaken neurotrophic responses [30]. Future research must persistently investigate the influence of exercise on BDNF levels in individuals with diabetes mellitus. This is similar to Cochrane systematic reviews, which found that exercise does not have a significant effect on cognitive health in older adults [31].

To enhance external validity, extensive RCTs must recruit participants from diverse age groups, ethnicities, and metabolic backgrounds. Standardized procedures for BDNF assessment, such as defining serum versus plasma, fasting status, and the timing of sample collection in relation to exercise, would improve comparability across trials [32]. Longer term interventions should include metabolic markers, neuroimaging, and neurocognitive measures to clarify the causal relationship between exercise, BDNF, and cognitive outcomes. Recent research shows that, in addition to structured exercise, lowering prolonged periods of inactivity is positively connected with plasma BDNF in patients with T2DM, independent of total moderate‐to‐vigorous physical activity.

This meta‐analysis overall concludes that regular exercise positively affects serum BDNF levels in diabetics, with increases observed in all age groups. Improvements in metabolic outcomes, including decreased body weight, BMI, and PBF, were often linked to BDNF increases. The regulation of BDNF is the primary and consistent benefit of exercise, as evidenced by the minimal or inconsistent effects it had on other neurotrophic factors (NGF, TrkB) and inflammatory markers (IL‐6, TNF‐*α*, and CRP). Overall, the results support the importance of exercise in fostering neuroplasticity and metabolic health, though it is unclear how it affects inflammation. Although the pooled results showed significance for the outcomes, the certainty of the results was variable for the measures. The certainty of the results for body fat, weight, and BDNF was rated as moderate for the outcomes, mainly due to imprecision and variability between the small number studies. The results suggest that the outcomes for FBG, body fat, weight, and BDNF need to be interpreted with caution, and further studies need to be done to further evidently confirm the results for the outcomes.

## 5. Strengths and Limitations

The included studies have several significant advantages. Compared with observational research, all four were randomized controlled trials, which improve methodological rigor and lower the possibility of bias. Their combined analysis of metabolic provided a more comprehensive view of the advantages of exercise for people with diabetes and obesity. Serum BDNF and, occasionally, other markers like NGF, TrkB, IL‐6, TNF‐*α*, and CRP were regularly measured in the studies, enabling investigation of both neurotrophic and inflammatory pathways. The inclusion of a variety of populations, from adolescents with Type 2 diabetes to older women with diabetes and depression and adults with the disease, offered important insights into how exercise affects various at‐risk groups throughout the lifespan. Additionally, the effects of the intervention were easier to interpret when standardized aerobic or combined aerobic‐resistance exercise protocols were used. Finally, one study included a detraining phase, providing novel proof of the endurance of exercise‐induced adaptations, a topic that is rarely covered in exercise trials. All these advantages raise the validity of the results and highlight how exercise may be used as a therapeutic approach to help people with diabetes and obesity achieve better metabolic health outcomes. In contrast to many exercise–BDNF trials, our included studies evaluated neurotrophic biomarkers in addition to depressive symptoms and functional endpoints, bridging the gap between biological plausibility and patient‐centered outcomes [33]. Our synthesis incorporates the most recent evidence from RCTs (2018–2023), including gender‐specific cohorts and newer modalities (NMES), which were not adequately represented in previous meta‐analyses.

This meta‐analysis has various limitations that should be noted when assessing the results. Firstly, despite conducting a thorough search across multiple databases some relevant studies might have been missed as retrieving all publications cannot be guaranteed with any search strategy. FBG was assessed in only two studies and a key limitation was the substantial heterogeneity observed across studies, particularly in the pooled analysis of FBG levels (*I* ^2^ = 94*%*) and the variability has also been contributed to by not standardizing units and assay methods. The marked differences in the magnitude of the reported effects limited the reliability and generalizability of the findings. Participant characteristics, intervention duration, differences in study design, exercise protocols, and outcome assessment methods may have contributed to this heterogeneity. Additionally, the stability and generalizability of the pooled estimates may have been affected by the inclusion of both parallel‐group and crossover randomized trials, along with the limited number of available studies. Publication bias could not be assessed due to a limited number of studies, which also makes the research less generalizable.

BDNF was evaluated using ELISA in all the studies, which does not distinguish between pro‐BDNF and mature BDNF, despite their distinct and occasionally competing biological activities. The studies had small sample sizes, which reduced statistical power and limited the generalizability of the findings. The intervention and follow‐up periods were brief, typically 12 weeks (about 3 months), with detraining effects investigated for only 8 weeks in one study; hence, the long‐term viability of exercise‐induced changes is unknown. Moreover, most trials were targeted entirely on BDNF, whereas other possible biological and psychological mediators, such as oxidative stress, hormonal regulation, or lifestyle factors, were either ignored or inadequately investigated. Research on NGF and TrkB in humans is limited, making it difficult to place the observed findings in a broader context. Furthermore, the populations investigated were restricted and specific, consisting of adults with Type 2 diabetes, women aged 50–70 with moderate depression, and Korean adolescents with obesity or T2DM. These restricted groupings limit the findings′ application to larger and more diverse populations. Differences in exercise duration, frequency, and intensity across research make direct comparison difficult and may explain certain outcome inconsistencies. Although exercise improved BDNF levels in some groups, the effects on other neurotrophic and inflammatory markers (NGF, TrkB, IL‐6, TNF‐*α*, and CRP) were inconclusive, preventing firm conclusions about the immunological impact of exercise in diabetes [34].

## 6. Clinical Implications

From a clinical perspective, it is possible that the integration of exercise programs into diabetes management interventions could be an economical and accessible means of supporting metabolic health. However, considering the small number of studies the results should be interpreted cautiously. In terms of global disease burden, it is possible that interventions such as exercise programs could be an important part of preventive metabolic care in diabetes and its complications.

## 7. Conclusion

In conclusion, this systematic review and meta‐analysis found that structured exercise programs may lead to an increase in serum BDNF levels and improve metabolic outcomes in patients with diabetes mellitus. The small number of trials and small sample sizes have limited the conclusions that can be drawn. Although these findings represent the potential role of exercise in modulating BDNF and therefore positive metabolic outcomes, its role in improving cognitive function still needs to be established. Further large‐scale randomized controlled trials are required to determine the long‐term neurological effects of exercise in patients with diabetes mellitus.

## Author Contributions

Rumeesa Parveen: conceptualization, methodology, validation, supervision, project administration, and writing—review and editing. Sara Jawaid: investigation, data curation, formal analysis, writing—original draft (Introduction), and writing—review and editing. Abdullah Amjad: investigation, data curation, writing—original draft (Results), and writing—review and editing. Araj Naveed Siddiqui: data curation, investigation, and writing—review and editing. M. Abdur Rahman Aizaz: data curation, investigation, writing—original draft (Methods and Abstract), and writing—review and editing. Syeda Sana‐e‐Zehra Naqvi: formal analysis, data interpretation, and writing—review and editing. Sarah Sohail: formal analysis, data interpretation, writing—original draft (Abstract and Discussion), and writing—review and editing. Dirha Amjad: formal analysis, writing—original draft (Results), and writing—review and editing. Umaimah Naeem: validation and writing—review and editing. Asma Azam: validation and writing—review and editing. Laiba Shamim: methodology, validation, formal analysis, and writing—review and editing. Sakan Binte Imran: methodology, validation, formal analysis, and writing—review and editing.

## Funding

No funding was received for this manuscript.

## Disclosure

All authors have read and approved the final version of the manuscript. Laiba Shamim had full access to all of the data in this study and takes complete responsibility for the integrity of the data and the accuracy of the data analysis.

## Ethics Statement

This study is a systematic review and meta‐analysis of previously published randomized controlled trials and does not involve collection of original data from human subjects or animals. All studies included were approved ethically by their institutional review boards, and informed consent was obtained from all participants. Ethical approval was not required for this review as this study involved synthesis and analysis of published literature that was publicly available and anonymized.

## Conflicts of Interest

The authors declare no conflicts of interest.

## Supporting Information

Additional supporting information can be found online in the Supporting Information section.

## Supporting information


**Supporting Information 1** File S1: Detailed search strategies and syntax used for database searching (PubMed, Scopus, ScienceDirect, Cochrane Central, and Embase).

**Supporting Information 2** File S2: Sensitivity analysis forest plot for brain‐derived neurotrophic factor (BDNF) and forest plot illustrating the effect of exercise interventions on fasting blood glucose levels compared with control groups in individuals with diabetes mellitus.

**Supporting Information 3**  

## Data Availability

Data sharing is not applicable to this article as no datasets were generated or analysed during the current study.
