# Supplementary material for: Association Between Exercise and Brain‐Derived Neurotrophic Factor (BDNF) Levels in Diabetes Mellitus: A Systematic Review and Meta‐Analysis
Source: J Diabetes Res. 2026 Oct 21;2026:5163895. doi: 10.1155/jdr/5163895 (PMC13686373; doi:10.1155/jdr/5163895)
Supplement: Supplementary file 1 [file JDR-2026-5163895-s001.docx]

**Search strategy for each database**

**Pubmed**

("Diabetes Mellitus"[Mesh] OR diabetes mellitus) AND ("Exercise"[Mesh] OR exercise OR physical exercise OR aerobic exercise OR resistance training OR combined exercise OR neuromuscular electrical stimulation OR NMES) AND ("Control Groups"[Mesh] OR control OR sedentary control OR placebo OR no intervention OR detraining) AND ("Brain-Derived Neurotrophic Factor"[Mesh] OR brain-derived neurotrophic factor OR BDNF)

Results: 65

**Scopus**

TITLE-ABS-KEY(("diabetes mellitus") AND (exercise OR "physical exercise" OR "aerobic exercise" OR "resistance training" OR "combined exercise" OR "neuromuscular electrical stimulation" OR NMES) AND (control OR "control group" OR "sedentary control" OR placebo OR "no intervention" OR detraining) AND ("brain-derived neurotrophic factor" OR BDNF))

Results: 98

**Science Direct**

("diabetes mellitus") AND ( "physical exercise" OR "aerobic exercise" OR "resistance training" OR "neuromuscular electrical stimulation" OR NMES) AND control AND ("brain-derived neurotrophic factor" OR BDNF)

Results: 854

**Cochrane central**

("diabetes mellitus") AND (exercise OR "physical exercise" OR "aerobic exercise" OR "resistance training" OR "combined exercise" OR "neuromuscular electrical stimulation" OR NMES) AND (control OR "control group" OR "sedentary control" OR placebo OR "no intervention" OR detraining) AND ("brain-derived neurotrophic factor" OR BDNF)

Results: 37

**Embase**

('diabetes mellitus'/exp OR 'diabetes mellitus':ti,ab,kw) AND ('exercise'/exp OR 'exercise therapy'/exp OR exercise:ti,ab,kw OR 'physical exercise':ti,ab,kw OR 'aerobic exercise':ti,ab,kw OR 'resistance training':ti,ab,kw OR 'combined exercise':ti,ab,kw OR 'neuromuscular electrical stimulation':ti,ab,kw OR NMES:ti,ab,kw) AND ('control group'/exp OR control:ti,ab,kw OR 'control group':ti,ab,kw OR 'sedentary control':ti,ab,kw OR placebo:ti,ab,kw OR 'no intervention':ti,ab,kw OR detraining:ti,ab,kw) AND ('brain derived neurotrophic factor'/exp OR 'brain-derived neurotrophic factor':ti,ab,kw OR BDNF:ti,ab,kw)

Results: 234
