# Supplementary material for: Association Between Exercise and Brain‐Derived Neurotrophic Factor (BDNF) Levels in Diabetes Mellitus: A Systematic Review and Meta‐Analysis
Source: J Diabetes Res. 2026 Oct 21;2026:5163895. doi: 10.1155/jdr/5163895 (PMC13686373; doi:10.1155/jdr/5163895)
Supplement: Supplementary file 2 [file JDR-2026-5163895-s002.docx]

**Sensitivity analysis BDNF**

**FBG (Forest plot illustrating the effect of exercise interventions on fasting blood glucose levels compared with control groups in individuals with diabetes mellitus)**
